# CONTEXTUAL FACTORS ASSOCIATED WITH DECREASED DAILY DEPRESSION AND ANXIETY AMONG DEMENTIA CAREGIVERS

**DOI:** 10.1093/geroni/igac059.399

**Published:** 2022-12-20

**Authors:** Frank Puga, Abigail Poe, Meghan Rafford, Danny Wang, Carolyn Pickering

**Affiliations:** The University of Alabama at Birmingham, Birmingham, Alabama, United States; The University of Alabama at Birmingham, Birmingham, Alabama, United States; The University of Alabama at Birmingham, Birmingham, Alabama, United States; The University of Alabama at Birmingham, Birmingham, Alabama, United States; The University of Alabama at Birmingham, Birmingham, Alabama, United States

## Abstract

Family caregivers of individuals with dementia (IWDs) have an increased risk of developing depression and anxiety. Little is known about daily protective factors that mitigate this risk. The purpose of this study was to identify everyday coping strategies used by family caregivers that reduce the daily odds of experiencing depression and anxiety-related symptoms. Daily diaries were used to examine whether pleasant non-care activities with the IWD, social connection, and exercise were associated with a decrease in depression and anxiety-related symptoms. A national sample of ADRD caregivers (N=165) completed diaries over 21-days (n=2,841). Participants were asked about their daily experiences as caregivers, coping strategies, and mental health. Data were analyzed using mixed-level modeling. Depression and anxiety symptoms were endorsed by 141 (85.45%) and 155 (93.94%) participants, respectively. Social connection was associated with a decrease in the daily odds of depression (OR: 0.71, CI: 0.54 – 0.94, p=0.016) and anxiety symptoms (OR: 0.74, CI: 0.56 – 0.97, p=0.032). Caregivers were also less likely to endorse depression-related symptoms on days when engaging in a pleasant non-care activity with the IWD was reported (OR: 0.70, CI: 0.52 – 0.93, p=0.015). Finally, anxiety-related symptoms were less likely to be endorsed on days when caregivers reported engaging in exercise (OR: 0.65, CI: 0.49 – 0.86, p=0.003). The results from this study help elucidate coping strategies that may mitigate the risk of depression and anxiety associated with caregiving. These findings inform potential components for interventions to help support the health and well-being of family caregivers of IWDs.

